# Valosin-Containing Protein (VCP)/p97 Expression Correlation of Prognosis of Clear Cell Renal Cell Carcinomas

**DOI:** 10.3390/biomedicines14040920

**Published:** 2026-04-17

**Authors:** Akgül Arıcı, Elif Akçay, Seda Ocaklı, Osman Demir, Fikret Erdemir

**Affiliations:** 1 Department of Medical Pathology, Tokat Gaziosmanpaşa University, 60030 Tokat, Türkiye; 2 Department of Histology and Embryology, Tokat Gaziosmanpaşa University, 60030 Tokat, Türkiye; 3 Department of Biostatistics, Tokat Gaziosmanpaşa University, 60030 Tokat, Türkiye; 4 Department of Urology, Tokat Gaziosmanpaşa University, 60030 Tokat, Türkiye

**Keywords:** renal cell carcinoma, VCP/p97, immunohistochemistry

## Abstract

**Background/Objectives**: Although certain established prognostic factors may occasionally fail to provide precise risk prediction in renal cell carcinoma (RCC), valosin-containing protein (VCP)/p97 has been implicated in a poor prognosis in various cancers, while its prognostic value in clear cell renal cell carcinoma (ccRCC) remains unknown. This study aimed to determine the independent prognostic value of VCP/p97 expression in ccRCC. **Methods**: This retrospective study included 137 ccRCC patients, and VCP/p97 expression was analyzed by immunohistochemistry and classified into either low or high expression based on the intensity of the staining in relation to the expression in endothelial cells. **Results**: High expression of VCP/p97 was significantly correlated with large tumor size (*p* < 0.001), Fuhrman nuclear grade (*p* = 0.003), advanced TNM stage (*p* < 0.001), and distant metastasis (*p* < 0.001). Kaplan–Meier analysis showed that the survival of patients with high expression of VCP/p97 was significantly reduced, and multivariate analysis revealed that high expression of VCP/p97 independently predicted poor survival (HR 2.09, 95% CI 1.06–4.15, *p* = 0.034) in addition to age, Fuhrman grade, and TNM stage. **Conclusions**: This study demonstrated that VCP/p97 expression, a newly identified prognostic factor, independently predicted a poor prognosis in ccRCC, and its expression may be a useful tool in identifying ccRCC patients with a poor prognosis.

## 1. Introduction

Renal cell carcinoma (RCC) accounts for 2–3% of all malignancies. More than 90% of all kidney malignancies are RCC [1]. According to global cancer statistics for 2022, among urological malignancies, RCC ranks third in incidence and mortality, after prostate and bladder cancers [2]. The incidence of RCC continues to increase by 1.5% each year [3]; it is reported as the sixth and the ninth most frequently diagnosed cancer in men and women, respectively [4]. The incidence is higher in men than in women, and the reported male–female ratio ranges from 1.5:1 to 2:1. RCC is a disease that affects older adults, most commonly seen in the 60–70-year age range [1]. Notably, the incidence of RCC in developed countries has more than doubled over the last half-century. This situation can be attributed to the increased use of radiological imaging methods, as well as to risk factors such as obesity and smoking [5,6]. Clear cell renal cell carcinoma (ccRCC) is the most common and biologically most aggressive histological subtype of renal cell carcinoma; it accounts for approximately 70–80% of all RCC diagnoses and is thought to originate from the epithelial cells of the proximal renal tubules. ccRCC is responsible for the vast majority of kidney cancer-related deaths [4,5,6,7].

The main prognostic factors in RCC include the TNM staging system and the Fuhrman nuclear grading system [7,8,9]. In RCC, the tumor stage at the time of diagnosis is the strongest determinant of survival. The TNM staging system, which includes primary tumor size and extent, lymph node involvement, and the presence of distant metastasis, remains the cornerstone of prognostic evaluation. While the 5-year survival rate is approximately 93% in patients with localized (Stage I) disease, it drops dramatically to approximately 12% in metastatic (Stage IV) cases. Increasing tumor size, especially in tumors exceeding 7 cm, is associated with more frequent adverse outcomes along with a higher nuclear grade and an increased probability of invasion into the renal sinus, perirenal fat tissue, or renal vein. Local extension into these structures is considered to be an indicator of aggressive biological behavior and is associated with decreased survival [5,8]. Characteristics determined as a result of pathological examination play a critical role in predicting the biological behavior of the disease. The Fuhrman grading system (Grades I–IV), which is based on the morphology of tumor cell nuclei, serves as an independent prognostic factor; high-grade (Grades III–IV) tumors are associated with poor survival [7,8]. The histological subtype also has prognostic significance; ccRCC is not only the most common subtype but also demonstrates higher metastatic potential compared to non-clear cell variants. The presence of tumor necrosis is an additional adverse pathological feature linked to unfavorable survival [5,10].

The current preferred treatment for RCC is surgery. Surgical resection, especially partial or radical nephrectomy, remains the cornerstone of curative treatment and is known to significantly improve overall survival [5,10]. In RCC, the clinical outcomes of patients with the same pathological stage and grade can be highly variable. Therefore, the identification of novel molecular biomarkers associated with tumor aggressiveness is of critical importance for accurate prognostic evaluation.

Valosin-containing protein (VCP), also known as p97, is a protein belonging to the AAA+ ATPase (ATPases Associated with diverse cellular Activities) family. Structurally, VCP/p97 forms a homohexameric barrel-like complex consisting of an N-terminal domain involved in substrate recognition, two ATPase domains (D1 and D2) responsible for oligomer assembly and hydrolysis, and a C-terminal tail [11,12]. VCP/p97 is a highly abundant protein that plays a critical role in the ubiquitin–proteasome system (UPS)—the main pathway controlling proteostasis—and accounts for approximately 1% of total cellular protein. Functioning as a molecular segregase, VCP/p97 uses the energy generated from ATP hydrolysis to unfold and extract ubiquitinated substrates from cellular organelles or protein complexes through its central channel, leading to the degradation of proteins via the 26S proteasome or autophagy. This activity is crucial for the maintenance of cellular homeostasis through the UPS; additionally, it governs critical pathways including endoplasmic reticulum-associated degradation (ERAD), mitophagy, lysophagy, and DNA repair mechanisms [13,14,15].

Cancer cells have higher protein synthesis rates and increased proteotoxic stress compared to normal cells; therefore, they are highly dependent on an active UPS and VCP/p97 [16,17]. Mounting evidence highlights a direct link between abnormal VCP/p97 expression and tumor progression, indicating that VCP/p97 plays a role as a potential carcinogenic marker for the progression and metastasis of various cancer types. VCP/p97 is frequently overexpressed in a wide range of malignancies, including colorectal, pancreatic, hepatocellular, and breast carcinomas. High VCP/p97 levels are consistently associated with aggressive clinicopathological features, metastasis, and poor patient prognosis [12,18,19,20,21]. Cancer cells utilize the segregase activity of VCP/p97 to mitigate the proteotoxic stress triggered by their high metabolic rates and rapid proliferation. Furthermore, VCP/p97 functions as a pro-survival factor by inhibiting apoptosis. Specifically, it sustains tumor growth by promoting the degradation of tumor suppressors such as p53 and the NF-*κ*B inhibitor I*κ*B*α*. Beyond survival, VCP/p97 also increases cell motility and invasive potential by modulating the actin cytoskeleton through the Rho-ROCK signaling pathway [16].

Although there is a substantial amount of literature on the prognostic value of VCP/p97 in different cancer types, VCP/p97 expression in RCC has been poorly investigated. To the best of our knowledge, there is limited information available regarding the association of VCP/p97 expression with conventional clinicopathological parameters in ccRCC [12,22]. In this study, we aimed to investigate the immunohistochemical expression of VCP/p97 and its independent prognostic value for survival in patients with ccRCC.

## 2. Materials and Methods

### 2.1. Patients

Our study included 145 cases diagnosed with ccRCC from radical nephrectomy material, based on histopathological examination, at the Department of Pathology, Faculty of Medicine, Tokat Gaziosmanpaşa University, between January 2010 and December 2022. Pathology reports of the cases were retrospectively reviewed by searching the pathology archive. Eight patients for whom archive material and/or clinical information could not be accessed during the search process were excluded from the study.

Clinicopathological data—including age, gender, tumor size, laterality, Fuhrman nuclear grade, tumor necrosis, lymphovascular invasion, renal capsule invasion, perirenal fat invasion, renal sinus invasion, renal vein invasion, Gerota’s fascia invasion, TNM stage, distant metastasis, and survival—for the 137 cases included in this study were obtained from hospital medical records and pathology reports. The study was approved by the local Tokat Gaziosmanpaşa University Faculty of Medicine Clinical Research Ethics Committee (Approval No: 24-KAEK-015).

### 2.2. Immunohistochemistry

Hematoxylin–eosin (H&E)-stained preparations from the cases were re-examined to confirm the diagnoses. For each patient, one formalin-fixed, paraffin-embedded block, containing sufficient tumor tissue and representing the highest Fuhrman nuclear grade of the tumor, was selected. Four-micrometer (4 μm)-thick paraffin sections were taken for immunohistochemical (IHC) analysis. The sections were stained with VCP/p97 antibody (ab11433, 1:500, Abcam, Cambridge, UK). IHC staining was performed with a Leica Bond-Max (Leica Biosystems, Nussloch, Germany) fully automated immunohistochemical staining device.

**Figure 1:**
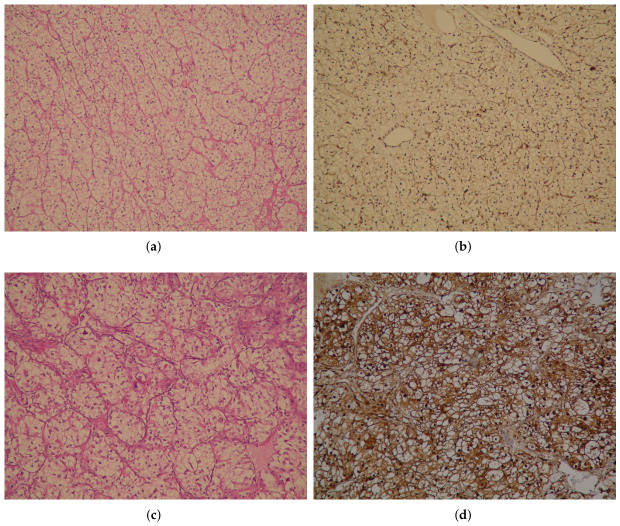
(**a**,**b**) Low valocin-containing protein (VCP/p97) expression in tumor cells. Weak VCP/p97 staining compared to endothelial cells. (**c**,**d**) High VCP/p97 expression in tumor cells. Strong VCP/p97 staining similar to endothelial cells (×100). (**a**,**c**) H&E; (**b**,**d**) VCP/p97.

The evaluation of IHC stains was performed by two pathologists who were unaware of the patients’ clinicopathological data. The evaluation was performed on ten randomly selected different fields (×100 magnification) on the sections. Cytoplasmic staining in tumor cells was considered positive staining. Positive staining in endothelial cells was used as an internal positive control. The staining intensity in the cytoplasm of tumor cells was compared with the staining intensity of endothelial cells. In cases showing heterogeneous staining, the dominant staining pattern was recorded. Absence of staining (score 0) or weaker staining than that of endothelial cells (score 1) was categorized as “low VCP/p97 expression”, while equal (score 2) and more intense staining than that of endothelial cells (score 3) was categorized as “high VCP/p97 expression” (Figure 1).

### 2.3. Statistical Analysis

Statistical analyses were performed using SPSS 27 (IBM SPSS Statistics for Windows, Version 27.0. Armonk, NY, USA: IBM Corp). Categorical variables were compared using the chi-squared test or Fisher’s exact test, while continuous variables were compared using Student’s *t*-test or the Mann–Whitney U test, as appropriate. Survival curves were constructed using the Kaplan–Meier method, and differences between groups were analyzed using the Log-rank test. Univariate and multivariate Cox proportional hazard regression analyses were performed to identify independent prognostic factors. A *p*-value less than 0.05 was considered statistically significant.

Overall survival (OS) was defined as the time from the date of nephrectomy to the date of death or the date of the last data collection (June 2025). Patients’ survival status and exact dates of death were verified through official national population registers integrated into the hospital system. There were no patients lost to follow-up in terms of survival status.

## 3. Results

### 3.1. Clinicopathological Characteristics of Patients

This study included 137 patients diagnosed with ccRCC: 97 males (70.8%) and 40 females (29.2%). The mean age of the patients was 61.2 ± 10.8 years, and the mean diameter of their tumors was 6.3 ± 2.7 cm. With respect to the Fuhrman grade of the tumors, Grade II was the most common, occurring in 64 patients (46.7%). Grade I accounted for 37 patients (27.0%), Grade III accounted for 31 patients (22.6%), and Grade IV accounted for 5 patients (3.6%). With respect to the TNM classification system, the majority of patients in the study were classified as having Stage I ccRCC, which accounted for 79 patients (57.7%). Stage II accounted for 13 patients (9.5%), Stage III accounted for 36 patients (26.3%), and Stage IV accounted for 9 patients (6.6%). Histopathologically, necrosis in the tumors was identified in 45 patients (32.8%), and lymphovascular invasion in the tumors was identified in 23 patients (16.8%). Local invasion in the tumors included renal capsule invasion in 37 patients (27.0%). Invasion in the perirenal fat tissue and the renal sinus was identified in 41 patients (29.9%) and 35 patients (25.5%), respectively. Invasion in the renal vein and in Gerota’s fascia was identified in 24 patients (17.5%) and in only 6 patients (4.4%), respectively. Distant metastasis was identified in 18 patients (13.1%).

### 3.2. Immunohistochemical Expression of VCP/p97 and Correlation with Clinicopathological Features

IHC analysis of the tumor tissue revealed the presence of VCP/p97 in the cytoplasm of the tumor cells. The patients were divided into two groups—those with low VCP/p97 expression levels (n = 64, 46.7%) and those with high VCP/p97 expression levels (n = 73, 53.3%)—based on the staining intensity in comparison to the endothelial cells.

The statistical correlations of VCP/p97 expression levels with clinicopathological variables are presented in Table 1. With regard to the demographic characteristics, there was no statistically significant difference in age between the group with low VCP/p97 expression levels (61.4 ± 10.9 years) and the group with high VCP/p97 expression levels (60.9 ± 10.8 years). However, high levels of VCP/p97 expression were significantly associated with male gender compared to female gender (64.9% vs. 25.0%, *p* < 0.001).

With regard to the tumor characteristics, high levels of VCP/p97 expression were significantly associated with large tumor size (*p* < 0.001). The mean tumor diameter was significantly higher in the high-VCP/p97-expression group (7.5 ± 2.9 cm) compared to the low-expression group (4.8 ± 1.4 cm). VCP/p97 overexpression was significantly correlated with high Fuhrman nuclear grade (*p* = 0.003). In the low-VCP/p97-expression group, the majority of the patients presented with low-grade tumors (Grade I–II: 88.7%), whereas in the high-expression group, a substantial percentage of the patients presented with high-grade tumors (Grade III–IV: 38.7%). In addition, a statistically significant association was found between high VCP/p97 expression levels and high TNM stages (*p* < 0.001). In the high-VCP7p97-expression group, a high percentage of the patients presented with high-stage tumors (Stage III–IV: 56.0%), whereas in the low-expression group, only a small percentage of the patients presented with high-stage tumors (Stage III–IV: 4.8%).

Further analysis of invasive features showed that high VCP/p97 expression was significantly correlated with lymphovascular invasion (*p* = 0.002), renal capsule invasion (*p* < 0.001), perirenal fat invasion (*p* < 0.001), renal sinus invasion (*p* < 0.001), and renal vein invasion (*p* < 0.001). In addition, invasion of Gerota’s fascia was only noted in the high-VCP/p97-expression group (n = 6), showing statistical significance (*p* = 0.016). Distant metastasis was noted in patients with high VCP/p97 expression (24.7%) more often than in those with low VCP/p97 expression (0.0%; *p* < 0.001). In contrast, no statistical correlation was noted between VCP/p97 expression and tumor necrosis (37.0% in high VCP/p97 expression, and 28.1% in low VCP/p97 expression; *p* = 0.215).

### 3.3. Survival Analysis

The median follow-up period for the cohort was 60 months (range: 1–161 months). During the follow-up period, 64 out of 137 patients (46.7%) died (event), and 73 patients (53.3%) were censored as they were still alive at the time of data analysis. Survival status was available for all patients.

**Figure 2:**
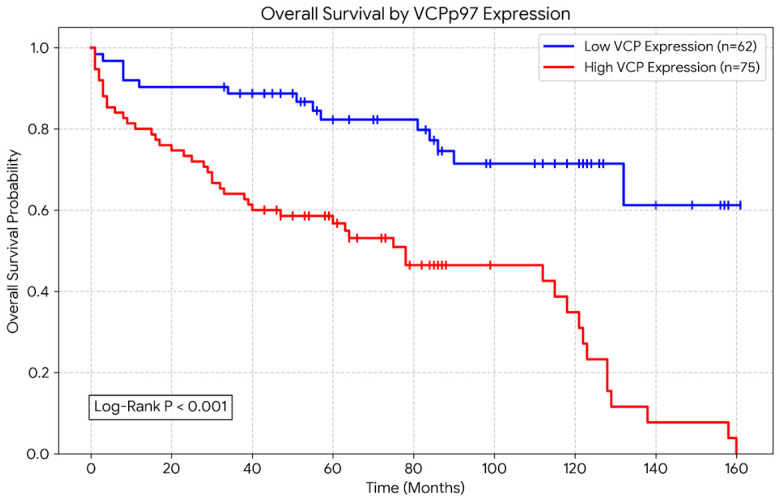
Kaplan–Meier survival curves for overall survival according to VCP/p97 expression in patients with clear cell renal cell carcinoma (ccRCC). Patients with high VCP/p97 expression showed significantly worse overall survival compared to those with low expression (Log-rank test, *p* < 0.001).

To determine the prognostic value of VCP/p97, Kaplan–Meier survival curve analysis was carried out, as depicted in Figure 2. The results clearly demonstrated that patients with high VCP/p97 expression levels had significantly reduced overall survival compared to those with low levels of VCP/p97 expression. This was determined to be statistically significant, with the Log-rank test showing *p* < 0.001. The survival curve clearly demonstrated the divergence in the survival patterns, with the high-expression cohort showing a sharp decline in survival probability over the study period.

### 3.4. Univariate and Multivariate Prognostic Analyses

To determine the independent prognostic factors for overall survival, univariate and multivariate Cox proportional hazard regression analyses were performed, as indicated in Table 2. In the univariate analysis, increased age (*p* < 0.001), high Fuhrman nuclear grade (III–IV vs. I–II; HR: 3.68, 95% CI: 2.12–6.37, *p* < 0.001), increased TNM stage (III–IV vs. I–II; HR: 4.10, 95% CI: 2.48–6.79, *p* < 0.001), and high VCP/p97 expression (HR: 3.78, 95% CI: 2.11–6.77, *p* < 0.001) were identified as prognostic factors for poor overall survival.

To further investigate the prognostic role of VCP/p97, adjusting for age, Fuhrman nuclear grade, and TNM stage, a multivariate analysis using the Cox regression model was performed. The multivariate analysis revealed high VCP/p97 expression as an independent adverse prognostic factor for overall survival (HR: 2.09, 95% CI: 1.06–4.15, *p* = 0.034). In addition, age (*p* < 0.001), Fuhrman nuclear grade (*p* = 0.002), and TNM stage (*p* < 0.001) were confirmed as independent prognostic factors in the multivariate analysis.

## 4. Discussion

RCC is a heterogeneous tumor characterized by aggressive behavior and metastatic potential, accounting for 90% of all kidney cancer cases [7]. ccRCC, the most common histological subtype, has a highly unpredictable nature in terms of clinical course [6]. In current clinical practice, the TNM staging system and Fuhrman nuclear grading system are primarily utilized to determine patient prognosis and guide therapeutic strategies [8]. The observation of differences in survival times and metastatic potential even among patients with the same anatomical stage and nuclear grade indicates that existing prognostic parameters may be insufficient for individual risk assessment [9]. In this context, the discovery of novel molecular biomarkers that can reflect the biological aggressiveness of the tumor at the cellular level and be integrated into routine histopathological evaluation has become one of the most critical objectives of oncological research [12]. In the present study, we investigated the clinicopathological and prognostic significance of the expression of VCP/p97—one of the fundamental regulators of cellular protein homeostasis (proteostasis)—in ccRCC patients. Our findings demonstrated that high expression levels of VCP/p97 were significantly associated with adverse clinicopathological features and decreased survival in ccRCC patients. Furthermore, multivariate analysis revealed that the expression level of VCP/p97 is an independent prognostic factor, indicating its potential as a reliable biomarker in the future.

VCP/p97 is one of the most evolutionarily conserved and highly abundant members of the AAA+ ATPase protein family in the cell [15]; its primary function is to participate in UPS and ERAD pathways, facilitating the transport and degradation of misfolded or damaged proteins by proteasomes [14]. The role of the UPS is critical in maintaining cellular protein homeostasis. Due to their uncontrolled proliferation and increased metabolic activities, cancer cells are constantly subjected to high levels of proteotoxic stress; in order to tolerate this stress and evade apoptosis, they are highly dependent on the scavenging and pro-survival functions of VCP/p97 [23]. Indeed, when our overall cohort data are examined, the detection of high-level VCP/p97 expression in 53.3% of ccRCC cases suggests that VCP/p97 plays a central role in the biological survival mechanisms of these tumors.

When we analyzed age and gender variables in terms of our demographic findings, as shown in Table 1, no statistically significant difference was found between the low- and high-VCP/p97-expression groups with respect to the mean age of the patients (61.4 and 60.9 years, respectively) (*p* = 0.749). This situation suggests that the genetic mutation profile of the tumor (e.g., VHL mutations), rather than cellular aging, dominates VCP/p97 regulation. On the other hand, in terms of gender distribution, high VCP/p97 expression was found to be statistically significantly more frequent in male patients compared to females (64.9% vs. 25.0%, *p* < 0.001). Epidemiological data indicate that RCC occurs approximately twice as frequently in men compared to women and can exhibit a more aggressive clinical course [5]. This observed gender difference may point to possible interactions of VCP/p97 expression with androgen receptor pathways. Indeed, in studies on prostate cancer, VCP/p97 has been reported to show a strong correlation with androgen-independent growth and tumor progression [24]. Based on this male-predominant high VCP/p97 expression that we detected in ccRCC, future research could be conducted on how gender-specific hormonal differences influence proteotoxic stress responses within the tumor microenvironment.

Tumor size is one of the most classic parameters reflecting tumor burden and proliferative capacity in RCC [8]. In our study, the mean tumor diameter in the high-VCP/p97-expression group was found to be significantly larger compared to the low-expression group (7.5 ± 2.9 cm vs. 4.8 ± 1.4 cm, *p* < 0.001). It is known that VCP/p97 accelerates the cell cycle, contributes to DNA damage repair mechanisms, and thereby enables the tumor to grow rapidly [13]. This finding of ours is consistent with the results of Yamamoto et al., who found a positive correlation between VCP/p97 expression and tumor diameter in gastric carcinoma [25]. This role of VCP/p97 in promoting tumor growth can be explained by its acceleration of the degradation of tumor-suppressor genes within the cell, such as p53 [26].

One of the most striking findings of our study in terms of histopathological differentiation parameters is the direct and significant relationship between the Fuhrman nuclear grade and VCP/p97 expression. According to our data, while the vast majority of patients in the low-VCP/p97 group (88.7%) had Grade I–II tumors, the rate of Grade III–IV was found to be 38.7% in tumors exhibiting high VCP expression (*p* = 0.003). As the Fuhrman grade increases, the nuclear atypia, pleomorphism, and mitotic activity of the cells also increase [9]. High-grade, poorly differentiated cancer cells are under immense ER stress due to massive protein synthesis. This indicates that dedifferentiated aggressive tumor cells have a greater need for VCP/p97 in order to survive and escape the toxicity of the mutant protein aggregates that they produce. In a study of 136 patients diagnosed with prostate cancer by Tsujimoto et al., a statistically significant relationship was identified between VCP/p97 and the Gleason score, which reflects the tumor grade. While high VCP/p97 expression was detected at a rate of 38.1% in tumors with a Gleason score of ≤6 (low-grade), this rate was found to be 90.4% in those with a Gleason score of 7, and 97.4% in those with a Gleason score of 8–10 (high-grade) [24]. In another study conducted on esophageal squamous cell carcinoma, tumors were grouped according to their histological differentiation grades, and the rate of high VCP/p97 was found to be higher in poorly differentiated tumors compared to well-differentiated ones [27].

In our study, no statistically significant difference was detected between the high- and low-VCP/p97 groups in terms of tumor necrosis (37.0% vs. 28.1%, *p* = 0.215). This situation may seem surprising. Necrosis is generally an indicator of aggressive and rapidly growing tumors. However, this non-significant correlation might stem from the fact that VCP/p97 is an anti-apoptotic and cytoprotective molecule. Hypoxia is the primary factor triggering necrosis in cancer tissue. It has been shown that VCP/p97 enhances the resistance of tumor cells to hypoxia by interacting with adaptor proteins (e.g., UBXD7) during the ubiquitination process of hypoxia-inducible factor 1-alpha (HIF-1alpha) [11]. Therefore, tumor cells with high VCP/p97 might have acquired a survival advantage, escaping necrosis even under hypoxic stress. This condition may biologically explain why there is no linear increase between the necrosis rate and VCP/p97.

The TNM stage and invasion parameters, which indicate the local and systemic spread of RCC, constitute the most important aspects of this study. VCP/p97 expression shows a parallelism with the TNM stage (*p* < 0.001). While the rate of Stage III–IV tumors was only 4.8% in the low-expression group, this rate reached 56.0% in the high-VCP group. Furthermore, when we analyzed invasion features, we established that lymphovascular invasion (*p* = 0.002), renal capsule invasion (*p* < 0.001), perirenal fat invasion (*p* < 0.001), renal sinus invasion (*p* < 0.001), and renal vein invasion (*p* < 0.001) showed a statistically strong correlation with the high-VCP/p97 group. Gerota’s fascia invasion, which constitutes important anatomical evidence of the tumor extending beyond the kidney, was observed exclusively in the high-VCP/p97-expression group across all six monitored cases (*p* = 0.016). This invasive phenotype may be directly related to the ability of VCP/p97 to regulate the epithelial–mesenchymal transition (EMT) [28]. In oral squamous cell carcinomas, it has been proven that VCP/p97 confers motility to the cancer cell by accelerating the degradation of adhesion molecules that maintain intercellular connections [29]. In a study conducted by Khong et al., it was shown that VCP/p97 regulates actin cytoskeletal dynamics, enabling tumor cells to form filopodia and penetrate tissue barriers [16]. Additionally, in hepatocellular carcinoma, VCP/p97 is known to promote the proliferation, migration, and invasion of liver cancer cells into surrounding tissues by activating the PI3K/AKT/mTOR pathway [30].

Looking at the distant metastasis rates, while no metastasis was detected in any patient in the low-VCP/p97 group (0%), it was observed that 24.7% of patients in the high-VCP/p97 group developed distant metastasis either at diagnosis or during follow-up (*p* < 0.001). This suggests that VCP/p97 may be associated not only with proliferative but also with pro-metastatic mechanisms in ccRCC. In the very recent literature, a direct molecular relationship controlling the development and progression of cholangiocarcinoma has been identified between VCP/p97 and the BAP1 protein. It has been stated that VCP/p97 directly binds to the tumor-suppressor BAP1 protein, facilitating its ubiquitin-dependent degradation. BAP1 is normally a potent tumor suppressor that inhibits cell proliferation and drives cancer cells into apoptosis. VCP/p97, present at high levels in cancer cells, constantly subjects BAP1 to degradation. Thus, cancer cells escape the suppressive effects of BAP1. This situation leads to the inhibition of apoptosis and the uncontrolled proliferation of cancer cells [31]. BAP1 mutations and BAP1 loss have been reported to be associated with aggressive clinical features, high risk of metastasis, and poor prognosis in ccRCC [6]. In the present study, the increased metastasis rate observed with high VCP/p97 levels may have been related to VCP/p97 mediating the degradation of the BAP1 protein. In light of data from the literature, this could be considered a possible hypothesis to explain our findings. Therefore, further studies are needed to elucidate the role of the VCP-BAP1 axis in ccRCC’s pathogenesis.

The most important aspect of the prognostic and analytical power of our study lies in our survival analyses. In the Kaplan–Meier survival curves (Figure 2), it can be observed that the curve of patients with high VCP/p97 expression diverges dramatically downwards, and their overall survival is statistically significantly shortened (Log-rank test, *p* < 0.001). Cox regression analyses (Table 2), which we applied to test whether VCP/p97 was overshadowed by other strong clinical parameters rather than relying solely on descriptive data, confirmed our hypothesis. In the univariate analysis, as expected, advancing age (HR: 1.06, *p* < 0.001), high Fuhrman grade (HR: 3.68, *p* < 0.001), advanced TNM stage (HR: 4.10, *p* < 0.001), and high VCP/p97 expression (HR: 3.78, *p* < 0.001) were found to have a significant negative impact on survival. Whether VCP/p97 independently holds significance, irrespective of stage and grade, was evaluated via multivariate analysis. After adding known prognostic factors of ccRCC—such as age, Fuhrman grade, and TNM stage—to the model and performing adjustments, high VCP/p97 expression maintained its statistical significance (*p* = 0.034). The expression level of VCP/p97 was proven to be an independent prognostic factor, independently increasing the mortality risk by 2.09-fold (HR: 2.09, 95% CI: 1.06–4.15). This finding of ours is similar to the results of studies that defined VCP/p97 as an independent adverse prognostic factor in colorectal cancer, thyroid cancer, breast cancer, and non-small-cell lung cancer [18,21,26,32,33]. This supports the notion that VCP is a universal malignancy indicator in epithelial-derived solid tumors.

Although classic parameters such as TNM staging and tumor size strongly predict survival in RCC, they may be insufficient on their own to explain the heterogeneity in the clinical course of patients. At this point, adding VCP/p97 immunohistochemical staining to routine pathological evaluation can reflect not only anatomical spread but also the biological aggressiveness of the tumor at the molecular level. The demonstration in our multivariate analysis that VCP/p97 is a prognostic factor independent of TNM stage and Fuhrman grade shows that this marker adds predictive value in addition to standard clinical parameters. Thus, it can help in the earlier detection of patients who appear anatomically low-risk but show high VCP/p97 expression at the cellular level and carry a risk of metastasis/recurrence. Patients’ follow-up and treatment strategies can be planned individually.

Due to its key role in protein degradation, homeostasis, and various cellular processes, VCP/p97 is utilized as a highly important and potential therapeutic target in the treatment of cancer, viral infections, and rare genetic diseases [12]. Recent studies have demonstrated that specific VCP/p97 inhibitors, such as CB-5083 and NMS-873, clog the “garbage disposal” system in cancer cells under high proteotoxic stress, triggering a fatal unfolded protein response and leading to selective cancer cell death [23]. Furthermore, other recent articles report that VCP inhibitors not only kill the cell but also disrupt the microtubule and cytoskeletal dynamics of the tumor, and that low-toxicity new-generation molecules capable of halting even viral replications have been developed [34,35,36]. Advanced experimental and clinical studies are needed regarding the potential of new-generation VCP inhibitors as ideal targets in the event that ccRCC patients develop resistance to current standard therapies.

Our study has certain limitations. First, our research followed a single-center and retrospective design, encompassing a relatively limited patient cohort (n = 137). Our findings have not been cross-validated with independent external datasets, and our results need to be validated in larger, multi-center, and prospective patient series. Second, although the immunohistochemical evaluation was performed using a semi-quantitative and standardized method, with endothelial cells serving as an internal control, it inherently may carry some degree of observer-dependent subjectivity. Finally, our patient cohort covered a long period between 2010 and 2022, so the different systemic treatment regimens received by the patients may show heterogeneity, which was not included in our analysis. The potential confounding effect on survival is a limitation of our study. Despite these limitations, our study presents a multivariate survival analysis in a homogeneous ccRCC group, adjusted for strong clinical confounders such as age and stage.

## 5. Conclusions

In conclusion, to the best of our knowledge, this study is the first in the literature to demonstrate the independent prognostic value of VCP/p97 expression in ccRCC patients. Our findings have shown that high VCP/p97 expression is strongly associated with larger tumor size, high Fuhrman nuclear grade, advanced TNM stage, and extensive local invasion features. The increased frequency of distant metastasis and the shortened overall survival time in the high-expression group clearly highlight the role of this protein in the biological aggressiveness of the tumor. Our multivariate analysis results prove that VCP/p97 can be used alone as a reliable poor prognostic factor, without being overshadowed by classical parameters such as age and stage. From a clinical perspective, these data indicate that integrating VCP/p97 into routine histopathological evaluation could be a valuable tool in the early detection of high-risk patients and the determination of individualized follow-up strategies. Furthermore, this dependence of cancer cells against proteotoxic stress paves a rational ground for the use of next-generation VCP/p97 inhibitors in ccRCC cases that develop resistance to standard therapies. However, due to the retrospective and single-center design of our study, the promising data obtained here should be interpreted with caution; they must be validated by multi-center and prospective studies with larger cohorts. In summary, VCP/p97 emerges as both a strong risk-determining biomarker in the management of ccRCC and a potential target for future oncological treatments.

## Figures and Tables

**Table 1:** Clinicopathological characteristics of patients, and their relationship with VCP/p97 expression.

Variables	Total (n = 137)	Low VCP (n = 64)	High VCP (n = 73)	*p*-Value
Age (years), mean ± SD	61.2 ± 10.8	61.4 ± 10.9	60.9 ± 10.8	0.749 ^a^
Gender, n (%)				<0.001 ^b^
Male	97 (70.8%)	34 (53.1%)	63 (86.3%)	
Female	40 (29.2%)	30 (46.9%)	10 (13.7%)	
Tumor Size (cm), mean ± SD	6.3 ± 2.7	4.8 ± 1.4	7.5 ± 2.9	<0.001 ^a^
Fuhrman Nuclear Grade, n (%)				0.003 ^b^
Grade I	37 (27.0%)	21 (33.9%)	16 (21.3%)	
Grade II	64 (46.7%)	34 (54.8%)	30 (40.0%)	
Grade III	31 (22.6%)	7 (11.3%)	24 (32.0%)	
Grade IV	5 (3.6%)	0 (0.0%)	5 (6.7%)	
TNM Stage, n (%)				<0.001 ^b^
Stage I	79 (57.7%)	59 (95.2%)	20 (26.7%)	
Stage II	13 (9.5%)	0 (0.0%)	13 (17.3%)	
Stage III	36 (26.3%)	3 (4.8%)	33 (44.0%)	
Stage IV	9 (6.6%)	0 (0.0%)	9 (12.0%)	
Tumor Necrosis, n (%)				0.215 ^b^
Absent	92 (67.2%)	46 (71.9%)	46 (63.0%)	
Present	45 (32.8%)	18 (28.1%)	27 (37.0%)	
Lymphovascular Invasion, n (%)				0.002 ^b^
Absent	114 (83.2%)	60 (93.8%)	54 (74.0%)	
Present	23 (16.8%)	4 (6.3%)	19 (26.0%)	
Renal Capsule Invasion, n (%)				<0.001 ^b^
Absent	100 (73.0%)	57 (89.1%)	43 (58.9%)	
Present	37 (27.0%)	7 (10.9%)	30 (41.1%)	
Perirenal Fat Invasion, n (%)				<0.001 ^b^
Absent	96 (70.1%)	59 (92.2%)	37 (50.7%)	
Present	41 (29.9%)	5 (7.8%)	36 (49.3%)	
Renal Sinus Invasion, n (%)				<0.001 ^b^
Absent	102 (74.5%)	58 (90.6%)	44 (60.3%)	
Present	35 (25.5%)	6 (9.4%)	29 (39.7%)	
Renal Vein Invasion, n (%)				<0.001 ^b^
Absent	113 (82.5%)	61 (95.3%)	52 (71.2%)	
Present	24 (17.5%)	3 (4.7%)	21 (28.8%)	
Gerota’s Fascia Invasion, n (%)				0.016 ^c^
Absent	131 (95.6%)	64 (100.0%)	67 (91.8%)	
Present	6 (4.4%)	0 (0.0%)	6 (8.2%)	
Distant Metastasis, n (%)				<0.001 ^c^
Absent	119 (86.9%)	64 (100.0%)	55 (75.3%)	
Present	18 (13.1%)	0 (0.0%)	18 (24.7%)	

^a^ Student’s *t*-test; ^b^ chi-squared test; ^c^ Fisher’s exact test.

**Table 2:** Univariate and multivariate Cox regression analysis for overall survival.

Variables	Univariate Analysis		Multivariate Analysis	
	HR (95% CI)	*p*-Value	HR (95% CI)	*p*-Value
Age (years)	1.06 (1.03–1.09)	<0.001	1.07 (1.04–1.10)	<0.001
Fuhrman Nuclear Grade				
Low (I–II) vs. High (III–IV)	3.68 (2.12–6.37)	<0.001	2.56 (1.42–4.61)	0.002
TNM Stage				
Low (I–II) vs. High (III–IV)	4.10 (2.48–6.79)	<0.001	3.36 (1.85–6.11)	<0.001
VCP/p97 Expression				
Low vs. High	3.78 (2.11–6.77)	<0.001	2.09 (1.06–4.15)	0.034

HR: hazard ratio; CI: confidence interval.

## Data Availability

The raw data supporting the conclusions of this article will be made available by the authors on request.
